# WeFaceNano: a user-friendly pipeline for complete ONT sequence assembly and detection of antibiotic resistance in multi-plasmid bacterial isolates

**DOI:** 10.1186/s12866-021-02225-y

**Published:** 2021-06-07

**Authors:** Astrid P. Heikema, Rick Jansen, Saskia D. Hiltemann, John P. Hays, Andrew P. Stubbs

**Affiliations:** 1 Department of Medical Microbiology and Infectious Diseases, Erasmus University Medical Center (Erasmus MC), Rotterdam, the Netherlands; 2 Department of Pathology, Clinical Bioinformatics Unit, Erasmus University Medical Center (Erasmus MC), Rotterdam, The Netherlands

**Keywords:** Oxford Nanopore technologies, MinIon, Plasmid assembly workflow, Anti-microbial resistance genes, Incompatibility factors, Flye

## Abstract

**Background:**

Bacterial plasmids often carry antibiotic resistance genes and are a significant factor in the spread of antibiotic resistance. The ability to completely assemble plasmid sequences would facilitate the localization of antibiotic resistance genes, the identification of genes that promote plasmid transmission and the accurate tracking of plasmid mobility. However, the complete assembly of plasmid sequences using the currently most widely used sequencing platform (Illumina-based sequencing) is restricted due to the generation of short sequence lengths. The long-read Oxford Nanopore Technologies (ONT) sequencing platform overcomes this limitation. Still, the assembly of plasmid sequence data remains challenging due to software incompatibility with long-reads and the error rate generated using ONT sequencing. Bioinformatics pipelines have been developed for ONT-generated sequencing but require computational skills that frequently are beyond the abilities of scientific researchers. To overcome this challenge, the authors developed ‘WeFaceNano’, a user-friendly Web interFace for rapid assembly and analysis of plasmid DNA sequences generated using the ONT platform. WeFaceNano includes: a read statistics report; two assemblers (Miniasm and Flye); BLAST searching; the detection of antibiotic resistance- and replicon genes and several plasmid visualizations. A user-friendly interface displays the main features of WeFaceNano and gives access to the analysis tools.

**Results:**

Publicly available ONT sequence data of 21 plasmids were used to validate WeFaceNano, with plasmid assemblages and anti-microbial resistance gene detection being concordant with the published results. Interestingly, the “Flye” assembler with “meta” settings generated the most complete plasmids.

**Conclusions:**

WeFaceNano is a user-friendly open-source software pipeline suitable for accurate plasmid assembly and the detection of anti-microbial resistance genes in (clinical) samples where multiple plasmids can be present.

## Background

The genome of bacteria can consist of both chromosomal and extra-chromosomal ‘plasmid’ DNA, which is freely present in the cytoplasm of the host bacterium. Dependent on the bacterial species, the size of the bacterial chromosomal ranges from 130 kbp [1] to over 14,000 kbp [2], whereas plasmids tend to be relatively much smaller with a size that varies between 1 kbp to more than 200 kbp [3]. These plasmids can be present as a single copy or in multiple copies within a single bacterium and replicate independently from the hosts’ chromosomal DNA. Further, several plasmids of different sizes may be present in a single bacterium.

The gene content of the genome varies greatly between bacterial species, but a repertoire of core genes that contain essential genetic information for living under ‘normal’ conditions is shared. The majority of core genes are located on the bacterial chromosome. However, bacterial plasmids may contain additional genes, including genes involved in virulence, plasmid mobility and antibiotic resistance, which may confer the bacterium with fitness advantages under certain conditions, e.g., during treatment with antibiotics. ‘Replicon’ genes, present on plasmids, promote and initiate plasmid replication and may be involved with plasmid ‘incompatibility’; the inability of different plasmids to co-exist stably in the same bacterial strain [4, 5]. Plasmids can be vertically transferred to daughter cells during bacterial replication and may be maintained through many generations via, for example, toxin-antitoxin systems [6]. Horizontal transfer of plasmids between the same (or different) bacterial species may also occur, facilitating the spread of antibiotic resistance genes and virulence factors through bacterial populations. As such, the detection, identification and tracking of plasmids within bacteria may allow interventions to be developed that lessen the impact of bacterial disease within human populations.

Whole-genome sequencing (WGS) for bacteria is a powerful tool that can be used for many applications, including the surveillance of bacteria in healthcare and environmental situations, as well as the detection of (novel) antibiotic resistance genes/mutations and the identification of genes involved in virulence. The success of WGS has led to an explosion in the number of publicly available bacterial genome sequences, yet less than 10% of these sequences are entirely closed single contig assemblies. (NCBI, February 2020, [7]).

The most widely utilized platform for performing WGS is the Illumina sequencing platform, which generates highly accurate sequence assemblies suitable for whole-genome gene analysis profiling and for the reliable identification of single nucleotide polymorphisms. However, the short read-length of Illumina reads (typically ~ 50–300 bp), makes the technology less suitable for the complete assembly of closed whole bacterial genomes. Further, complete assembly of plasmids is potentially challenging due to the presence of repeat sequences (homo- or heteropolymeric) of DNA that may be shared between plasmids or the plasmid and bacterial chromosome [8]. In contrast, long sequence reads (up to many thousands of nucleotides sequenced per sequencing read) may be generated by third-generation sequence platforms such as PacBio and Oxford Nanopore Technologies (ONT). Long read sequences potentially enable more accurate and complete assemblies of plasmids and bacterial chromosomes.

The low start-up cost, the small size of the sequencer (portability) and easy library preparation means that the ONT platform has quickly generated interest by microbiological researchers and diagnosticians. ONT has implemented easy to use software that is freely available and accessible; however, the complete assembly of ONT generated sequence reads still is difficult. The relatively high error rate (5–20% [9]) generally makes ONT reads incompatible with current popular and widely used software tools for sequence read assembly developed for low error rate Illumina sequencing. It is true that bioinformatics-based ONT assembly tools such as Miniasm [10] and Canu [11] have been developed, and accompanying scripts for these assembly tools are freely available. However, the implementation and use of these scripts require a certain level of bioinformatics knowledge that most non-bioinformaticians lack or do not have access to in healthcare and environmental situations. Alsoimportant is the option to visualize and evaluate the quality control parameters applicable for sequencing and assembly, as well as the ability to perform downstream data analysis. In conclusion, there is a current need for long-read sequence assembly and analysis pipelines that are accessible and easy-to-use for non-bioinformaticians interested in WGS of bacterial chromosomes and plasmids. We have developed WeFaceNano, an easy ‘to client’ application that is accessible via a web browser GUI that incorporates two fast long read sequence assemblers, quality control reporting, BLAST [12] database search function and an option to identify antibiotic resistance genes and plasmid incompatibility groups. WeFaceNano can be downloaded from GitHub as code and application [13].

### Implementation

**Fig. 1:**
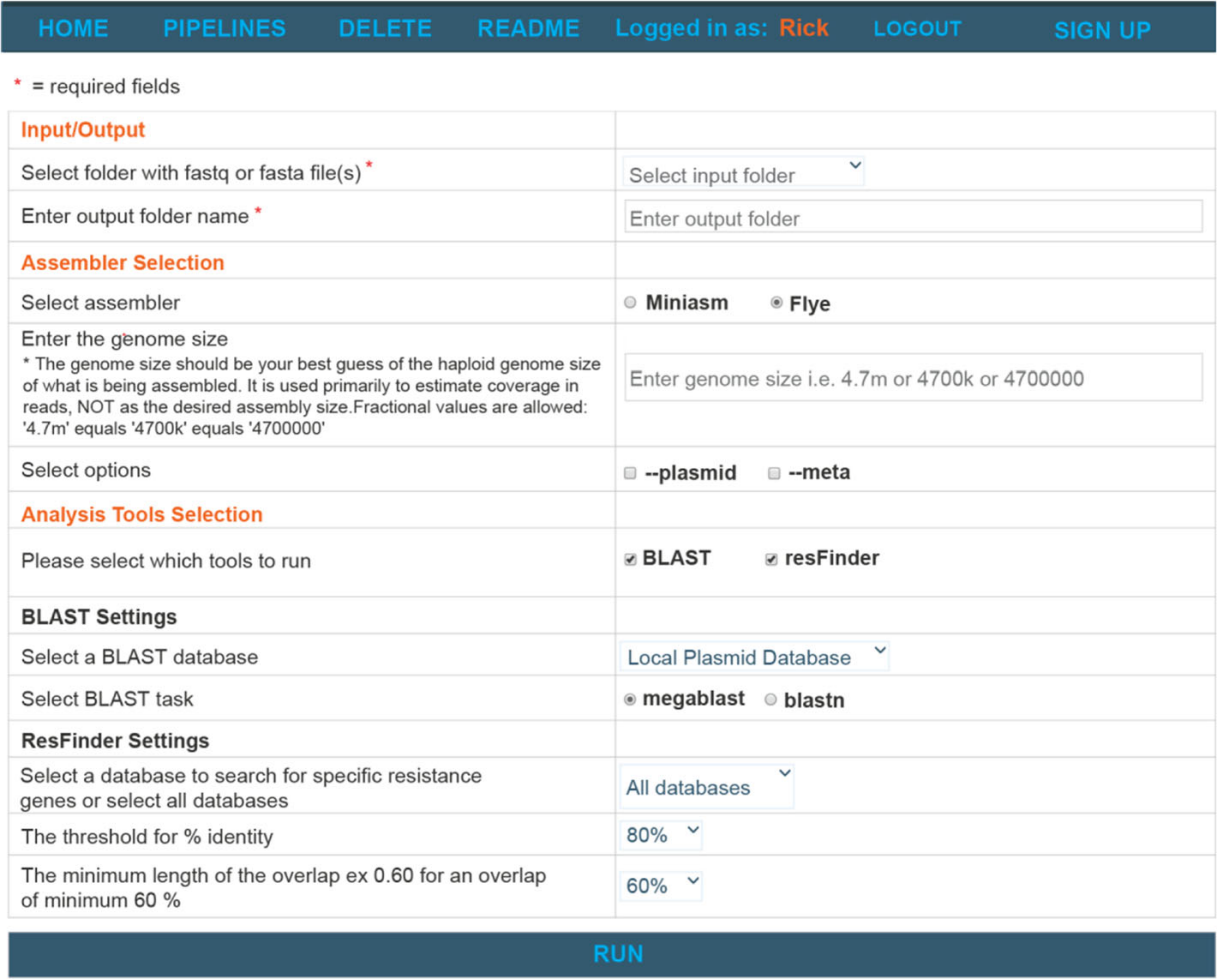
The WeFaceNano interface showing the available workflow tools and settings. Within the interface, the input folder containing the raw sequence data and output folder can be selected. There is a choice between two assemblers, including a selection for assembly settings when the Flye assembler is chosen. An estimated genome size has to be given, and there are options to do BLAST or ResFinder analysis with the possibility to provide desirable settings

**Fig. 2:**
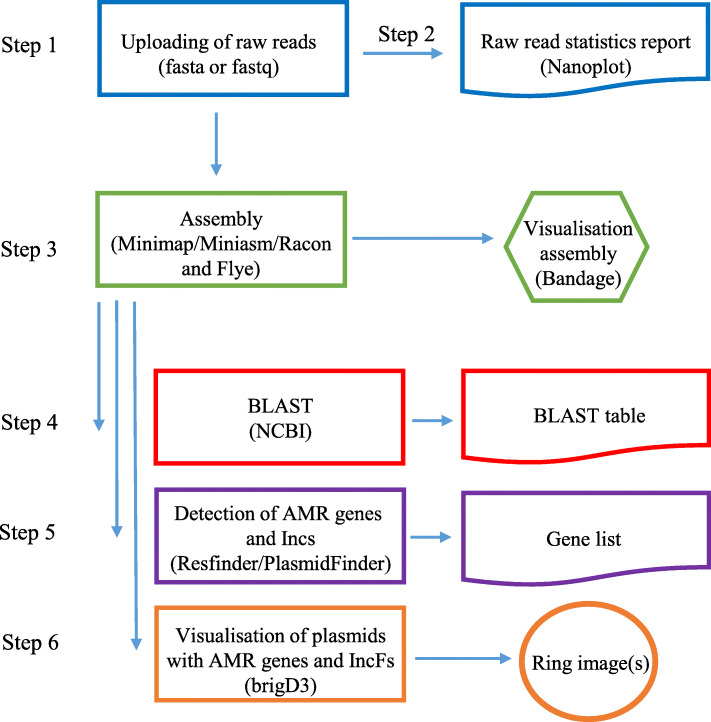
Outline of the WeFaceNano workflow, including the output and used software for each step. The steps of WeFaceNano include: (1) easy data upload, (2) a report with raw read statistics including histogram plots, (3) fast assembly and assembly visualizations, (4) BLAST identification of known plasmids summarized in a BLAST table, (5) detection of anti-microbial resistance genes and plasmid incompatibility factors summarized in an anti-microbial resistance gene table and an incompatibility factors Table. (6) web reporting, including ring image(s) of the results

WeFaceNano preferably should be installed on a server by a bioinformatician and with the option for users to upload raw sequence data. After this initial setup, WeFaceNano provides an easy-to-use web interface that was developed for research scientists with no programming or bioinformatics experience. The first page of the interface displays the main features of the application and gives access to the analysis tools (Fig. 1). The WeFaceNano workflow includes six steps: (1) data upload, (2) a report with raw read statistics, (3) fast assembly and visualizations, (4) BLAST identification of known plasmids, (5) detection of anti-microbial resistance genes and plasmid incompatibility factors and (6) web-based reporting including relevant visualisations of the results (see Fig. 2). For testing the application, a test dataset, including the output of the test dataset generated by WeFaceNano, is made available (https://github.com/ErasmusMC-Bioinformatics/WeFaceNano).

#### Step 1: data upload and storage of output

The WeFaceNano input data can be uploaded from a network drive, and the assembly and analysis results are stored on the same drive, in a folder assigned by the user.

#### Step 2: raw read statistics report

NanoPlot (v1.20.1, [14, 15]) was included for the generation of a read statistics report. The report contains a table with the following statistics: mean read length, median read length, the number of reads, read length N50 and total bases. The read statistics are also plotted as; a histogram with the read lengths, a histogram with the read lengths after log transformation, a weighted histogram with the read lengths, a weighted histogram with the read lengths after log transformation and the yield by length [16].

#### Step 3: assembly and visualisations

There are two sequence assemblers, Miniasm and Flye, available within WeFaceNano. The Miniasm assembler comprises the programs Porechop (v0.2.4, [17]), Minimap2 (v2.15-r915, [18, 19]), KmerGenie (v1.7051, [20, 21]), Miniasm (v0.3-r179, [10, 22]) and Racon (v1.3.3, [23, 24]). Porechop removes the adaptors from raw ONT reads. In Minimap2, the reads are aligned using optimal k-mer settings as identified by KmerGenie. All k-mer possibilities between k13-k21, a range that is optimal for Minimap2, are tested. Miniasm concatenates the aligned reads to an initial consensus sequence. Then, Racon starts a second round of alignment and assembly using the consensus sequence generated by Miniasm as a reference. This second round will result in a more accurate assembly and the generation of the final consensus contigs that are used for further analysis. Users have the option to eliminate contigs that are below an indicated basepair (bp) length.

The Flye (v2.4.2, [25, 26]) assembler automatically selects an optimal k-mer size based on an estimated genome size and has a default- (Flye) a meta- (metaFlye) and a plasmid- (plasmidFlye) option for assembly. Besides the default settings of Flye, metaFlye applies an algorithm that tries to generate longer contigs in order to assemble a complete genome, whereas plasmidFlye also includes smaller contigs (excluded in the metaFlye) in the assembly. It is possible to combine the meta- and the plasmid option to find additional contigs.

The program Bandage (v0.8.1, [27, 28]) is linked to the output of both assemblers and visualizes the generated contigs. Images generated by Bandage are stored as SVG files and can be viewed on the results page) [16] of the assembly section.

#### Step 4: BLAST identification of known plasmids

The BLAST function (v2.9.0+, [3]) allows submission of the assembled contigs to the remote NCBI Nucleotide collection (nr/nt) blast database, or to a locally installed database, for BLAST analysis. The BLAST functions megablast and blastn are available in WeFaceNano, which are used to determine which plasmids from the NCBI nucleotide or the reference plasmid database (http://gigadb.org/dataset/view/id/100387/Sample_page/2/File_sort/format_id) could be identified from the contigs generated from step 3.

#### Step 5: detection of antibiotic resistance genes and plasmid incompatibility factors

Anti-microbial resistance genes can be detected by a standalone local version of ResFinder (based on version 2.1, https://bitbucket.org/genomicepidemiology/resfinder/src/master/). In WeFaceNano all resistance gene databases or databases of choice can be selected. The following databases are included in WeFaceNano: aminoglycoside, beta-lactam, colistin, fosfomycin, fusidic acid, macrolide, nitroimidazole, oxazolidinone, phenicol, quinolone, rifampicin, sulphonamide, tetracycline, trimethoprim and glycopeptide. Thresholds for the percentage of identity and the minimum length of overlap can be pre-set. Anti-microbial resistance due to point mutations are not detected by the used version of ResFinder.

The PlasmidFinder database (v2.1, [12, 29]) was included to identify plasmid incompatibility factors. The Enterobacteriaceae database is currently installed in WeFaceNano, but other databases are available in the PlasmidFinder Bitbucket repository [30], which can be implemented in WeFaceNano.

#### Step 6: visualisation of BLAST, ResFinder and PlasmidFinder results

To visualise the BLAST results, anti-microbial resistance (AMR) genes and plasmid incompatibility factors, a custom version of brigD3 [31] was added to the WeFaceNano workflow. BrigD3 generates a ring image with an inner ring that reflects the top BLAST hit and outer rings that depict the contigs that match with the top BLAST hit. The presence and location of antibiotic resistance genes and plasmid incompatibility factors are indicated in different colours [16].

### Dataset used to test and validate the pipeline

Publicly available raw ONT sequence reads of plasmid DNA extracted from 12 bacterial strains were used to test and validate WeFaceNano. The plasmids were extracted as described from twelve multidrug-resistant bacterial strains (9x *E. coli*, 1x *S. typhimurium*, 1x *V. parahaemolyticus*, and 1x *K. pneumoniae* [32]. After Albacore (v1.0.3) base calling and de-multiplexing, a total of 21 plasmids were assembled using the Canu assembly tool and analyzed using PlasmidFinder [29], ResFinder [33] and ISfinder [13] databases and BLASTN. The raw ONT reads used to validate and test the pipeline, and Canu assemblies are available at: http://gigadb.org/dataset/100387

### Quality and completeness of the plasmid assemblies

To test and validate the WeFaceNano pipeline, published and publicly available raw ONT sequence reads of plasmid DNA extracted from 12 bacterial strains [32], were assembled with the Miniasm and Flye assemblers, and further analyzed using the WeFaceNano workflow. Subsequently, the WeFaceNano results were compared to the published assembly and analysis results (14). In general, similar results were obtained when the assemblies generated in WeFaceNano was compared to the published Canu assemblies, with 100% BLAST-mediated identification of the plasmids and 97.5% identity at 98.2% coverage for the Miniasm assembler and 97.6% identity at 95.1% coverage for the Flye assembler at default settings, respectively (Table 1).

**Table 1:** Top blast hit, coverage and identity of contigs assembled by the Miniasm and Flye assemblers. For the Flye assembler, several settings, default, meta and plasmid, were tested. The samples represent 12 plasmid DNA extracts that contained multiple multidrug resistance-encoding plasmids and were published previously. *, newly assembled with the Flye assembler using the meta option; **, average coverage and identity of plasmids that could be assembled and had a BLAST hit

Sample	Top BLAST hit	Size (bp)	Assembler Size (bp) coverage (%)/identity (%)			
			Miniasm	Flye	metaFlye	plasmidFlye
RB01	RB01-LZ135-CTX-128976	128,976	127,607 99/98.5	128,991 100/99.2	128,925 100/99.2	128,962 100/99.2
RB01	RB01-LZ135-NDM-90845	90,845	172,204 100/98.0	91,325 99/98.9	91,353 99/98.9	91,353 100/98.9
RB02	RB02-JN105-IncN-CTX-139496-N	142,307	143,553 99/96.3	117,855 100/96.7	144,482 99/96.6	117,851 81/96.7
RB02	RB02-JN105-IncF-TET-116277-N	116,277	119,228 99/97.9	119,956 99/97.8	119,986 99/97.8	119.936 99/96.7
RB02	RB02-JN105-IncY-CTX-98443	98,443	98,276 99/98.7	98,754 100/98.9	99,197 100/98.8	99,043 100/98.3
RB02	RB02-JN105-IncN-NDM6–55342	55,342	64,116 99/84.2	56,170 98/83.7	62,679 99/83.6	56,172 98/83.9
RB02	RB02-JN105-IncX-NDM5–45823	45,823	46,253 100/97.7	37,738 100/98.0	46,466 99/98.6	37,767 99/98.6
RB03	RB03-WH96T-IncF-OXA-153088	153,088	152,062 99/98.5	150,101 99/98.1	150,051 100/99.1	150,093 99/99.1
RB03	RB03-WH96T-IncN-NDM1–56215	56,215	58,008 100/98.5	52,703 98/98.9	56,496 100/98.9	52,711 98/98.9
RB04	RB04-SZ584-1 T-IncY-130,821	130,821	133,587 99/97.4	134,643 99/96.9	134,537 99/96.7	–
RB04	RB04-SZ584-1 T-IncF-TET-114056	114,065	111,330 99/98.5	114,409 100/99.2	114,405 100/99.0	114,434 100/98.9
RB04	RB04-SZ584-1 T-IncX3-NDM1-56 K-NC	55,919	214,172 99/95.4	10,965 10/NA	234,535 100/95.7	5,036 10/NA
RB04	RB04-234 K-Flye*	234,535	214,172 90/98.0	–	234,535 100/100	–
RB05	RB05-C267-IncA/C-CTX-166467	166,467	165,709 97/98.9	313,534 100/98.9	166,440 100/99.3	313,534 100/98.9
RB06	RB06-C499-IncA/C-CTX-192739	192,739	191,256 99/98.7	192,983 100/98.9	192,728 100/99.3	192,720 100/98.9
RB07	RB07-vb0506-IncA/C-CTX-133742	133,742	130,025 94/98.6	–	133,339 100/97.6	–
RB09	RB09-IncN-KPC-68571	68,571	133,828 99/98.6	68,899 99/98.9	68,904 99/98.9	68,895 99/98.9
RB10	RB10-29KPC-IncF-TET-136532	136,532	133,611 98/98.8	136,726 100/99.3	136,713 100/99.3	136,710 100/99.3
RB10	RB10-29KPC-IncY-KPC-98 K-N	95,908	96,533 95/98.5	97,091 99/98.1	97,602 99/98.1	97,528 99/98.2
RB11	RB11-IncF-IncHI-KPC-238153	238,153	238,205 99/98.4	255,475 100/98.8	229,070 100/98.8	239,791 100/98.8
RB12	RB12-74 T-KPC-IncF-115 K-N	115,689	117,851 98/98.0	118,528 99/97.8	111,151 94/97.8	118,574 99/97.8
RB12	RB12-74 T-KPC-IncN-IncX1-KPC-108 K-N	107,969	108,968 96/97.7	104,277 100/97.3	110,786 100/97.5	104,278 94/97.4
Average coverage/identity**			**98.2/97.5**	**95.1/97.6**	**99.4/97.7**	**93.5/97.7**

**Fig. 3:**
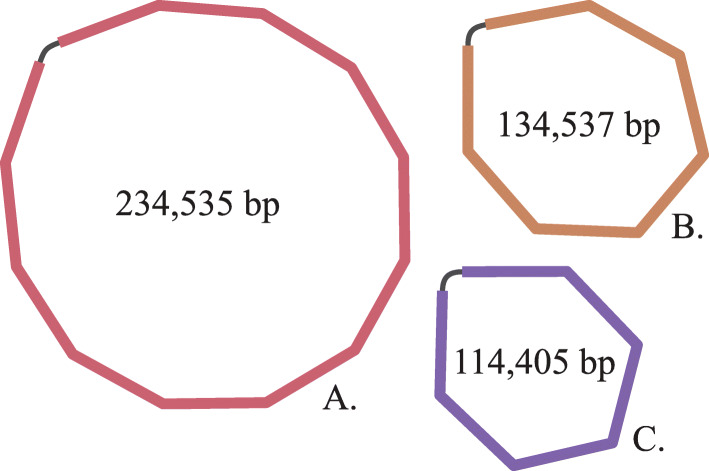
Bandage visualization of MetaFlye assembled plasmids of sample RB04. **A**. plasmid RB04-234 K-Flye. **B.** plasmid RB04-SZ584-1 T-IncY-130,821. **C. **plasmid RB04-SZ584-1 T-IncF-TET-114,056

MetaFlye is an option of the published Flye assembler software, which applies an algorithm that generates longer contigs. MetaFlye generated the highest identity and coverage values compared to the assemblers Miniasm, Flye with default settings and plasmidFlye, using data obtained from the reference plasmids. Interestingly, the use of metaFlye resulted in 3 complete circular plasmids (Fig. 3) in sample RB04 with sizes of 234,535 bp, 134,537 bp and 114,405 bp, compared to two circular plasmids with sizes of 130,821 bp and 114,065 bp and a linear plasmid fragment of 55,919 bp (plasmid RB04-SZ584-1 T-IncX3-NDM1-56 K-NC) when the Canu assembler was used. Apparently, the metaFlye assembler performed better in assembling a 234,535 bp plasmid, designated as RB04-234 K-Flye, in sample RB04.

Alignment of the 234,535 bp RB04-234 K-Flye plasmid against the published incomplete 55,919 bp linear fragment showed a coverage of 100% (Table 1), indicating that the incomplete 55,919 bp linear fragment was a segment of the complete 234,535 bp circular plasmid assembled by metaFlye. Further, previously performed pulsed-field gel electrophoreses of plasmid DNA of sample RB04 showed the presence of 3 bands, including a band with a size of 230 kbp [32], demonstrating that a plasmid with such size is present in sample RB04.

Flye with default settings did not result in the complete assembly of plasmid RB04-234 K-Flye. Instead, two circular contigs with sizes of 134,643 bp and 114,409 bp were generated (Table 1) that aligned against the 130,821 bp and 114,065 reference plasmids RB04-SZ584-1 T-IncY-130,821 and RB04-SZ584-1 T-IncF-TET-114056. The third contig that was assembled by Flye was a small 10,965 bp linear contig that only had 10% coverage when aligned against the linear plasmid fragment RB04-SZ584-1 T-IncX3-NDM1-56 K-NC.

PlasmidFlye failed to assemble four out of the twenty complete reference plasmids. Furthermore, in sample RB04, plasmidFlye generated an incomplete version (5,036 bp) of reference plasmid fragment RB04-SZ584-1 T-IncX3-NDM1-56 K-NC with a coverage of only 10% (Table 1).

Although the Miniasm assembler did assemble the two complete reference plasmids RB04-SZ584-1 T-IncY-130,821 and RB04-SZ584-1 T-IncF-TET-114056 of sample RB04 and a larger contig off 214,172 bp that contained the sequence of the linear plasmid fragment RB04-SZ584-1 T-IncX3-NDM1-56 K-NC, it was unable to generate the 234,535 kb plasmid RB04-234 K-Flye that was assembled by metaFlye.

MetaFlye was demonstrated to perform better when large repeats are present in a sequence [26]. We, therefore, assessed if such large repeats were present in the metaFlye assembled plasmid RB04-234 K-Flye, but this was not the case. Instead, four small transposase repeats were identified with sizes between 334 and 355 bp (data not shown). Although this is not expected due to the small size, it is possible that these transposase repeats hindered the complete assembly of plasmid RB04-234 K-Flye by the other assemblers.

The average top BLAST hit identity of the assembled plasmids was over 97% for all assemblers, with the Miniasm assembler having the lowest identity (97.42%) and metaFlye the highest identity (97.70%).

Minor differences in the sizes of the assemblies of the same plasmid were observed (Table 1) and are probably due to the difference in assemble algorithms combined with the sequence errors present in the nanopore raw reads. Compared to the references, large differences in the assembly sizes were observed in samples RB01 (RB01-LZ135-CTX-128976, Miniasm), RB04 (RB04-SZ584-1 T-IncX3-NDM1-56 K-NC, Miniasm and MetaFlye), RB05 (RB05-C267-IncA/C-CTX-166467, Flye and plasmidFlye) and RB09 (RB09-IncN-KPC-68571, Miniasm). In samples RB01, RB05 and RB09 these size differences were due to the same plasmid sequence being present with two copies within one contig. For the plasmid RB04-SZ584-1 T-IncX3-NDM1-56 K-NC it was unclear why a longer contig was produced by the Miniasm and Flye assemblers. It is possible that the assembled sequence of the reference plasmid is incomplete. 

### Detection of anti-microbial resistance (AMR) genes

The presence of AMR genes was assessed for all plasmids assembled with MetaFlye using the default settings for ResFinder in WeFaceNano. All previously described AMR genes were detected and 28 additional AMR genes were identified by WeFaceNano. Of the additionally identified AMR genes, 15 were detected in the 234,535 bp plasmid RB04-234 K-Flye compared to only two AMR genes in the incomplete plasmid that was published previously (Table 2). The remaining 13 additionally identified AMR genes were found in several other plasmids. The use of an updated version of ResFinder in WeFaceNano may have contributed to the identification of the additional AMR genes.

**Table 2:** Anti-microbial resistance genes and incompatibility factors identified in the assembled plasmids. *, a plasmid that could only be assembled with the Flye assembler using the meta option; anti-microbial resistance genes and incompatibility factors indicated in bold were additionally identified by WeFaceNano

Sample	Plasmid	Anti-microbial resistance genes	Incompatibility factors
RB01	RB01-LZ135-CTX-128976	aac(6′)-Ib; aadA5; blaCTX-M-15; **blaOXA-534**; **catB3**; dfrA17; mph(A); sul1; tet(A)	IncFIA
RB01	RB01-LZ135-NDM-90845	aadA2; blaNDM-5; **blaTEM-1B**; dfrA12; rmtB; sul1	IncFII
RB02	RB02-JN105-IncN-CTX-139496-N	aadA1; **aadA2**; aph(3′)-Ia; blaCTX-M-55; **blaTEM-1B**; cmlA1; dfrA12; met(B); rmtB; sul3	IncFII; IncN
RB02	RB02-JN105-IncF-TET-116277-N	aac(3′)-IIa; aadA5; dfrA17; mph(A); sul1; tet(A)	IncFII; IncFIA
RB02	RB02-JN105-IncY-CTX-98443	–	IncY
RB02	RB02-JN105-IncN-NDM6–55342	**blaNDM-1**; dfrA14; qnrS1	IncN
RB02	RB02-JN105-IncX-NDM5–45823	blaNDM-5	IncX3
RB03	RB03-WH96T-IncF-OXA-153088	aac(6′)-Ib-cr; **blaOXA-1**; **catB3**; tet(B)	IncFIA; IncFIC
RB03	RB03-WH96T-IncN-NDM1–56215	blaNDM-1; dfrA14	IncN
RB04	RB04-SZ584-1 T-IncY-130,821	–	IncY
RB04	RB04-SZ584-1 T-IncF-TET-114056	aadA1; **aadA2**; cmlA1; dfrA12; floR; sul2; tet(A); tet(M)	IncFIB; IncFIC
RB04	RB04-SZ584-1 T-IncX3-NDM1-56 K-NC	blaNDM-1; floR	IncX3
RB04	RB04-234 K-Flye*	**aph(3′)-la**; **aph(6)-ld**; **aac(6′)-lb-cr**; blaNDM-1; **blaCMY-2**; **blaOXA-1**; **blaDHA-1**; **mph(A)**; floR; **catB3**; **qnrB4**; **ARR-3**; **sul1**; **sul2**; **tet(A)**	**IncA/C2**; IncX3
RB05	RB05-C267-IncA/C-CTX-166467	aadA1; **ant(2″)-Ia**; **ARR-2**; blaCTX-M-55; blaOXA-10; catA2; cmlA1; floR; sul1; tet(A)	IncA/C2
RB06	RB06-C499-IncA/C-CTX-192739	aac(3)-Iid; aadA1; **ant(2″)-Ia**; blaCTX-M-55; blaOXA-10; cmlA1; floR; qnrA1; sul1; tet(A)	IncA/C2
RB07	RB07-vb0506-IncA/C-CTX-133742	**aph(3″)-Ib**; **aph(6)-Id**; **blaCTX-M-182**; dfrA23; tet(A)	IncA/C2
RB09	RB09-IncN-KPC-68571	aac(6′)-Ib-cr; ARR-3; blaCTX-M-3; blaKPC-2; blaTEM-1B; qnrS1	IncN
RB10	RB10-29KPC-IncF-TET-136532	aac(3)-IId; aadA5; **aph(3″)-Ib**; **aph(6)-Id**; blaTEM-1B; dfrA17; mph(A); qepA1; rmtB; sul1; sul2; tet(A)	Inc FIA; IncFIB; IncFII
RB10	RB10-29KPC-IncY-KPC-98 K-N	blaKPC-2	IncY
RB11	RB11-IncF-IncHI-KPC-238153	blaKPC-2; dfrA14	IncFIB
RB12	RB12-74 T-KPC-IncF-115 K-N	–	Col156; IncFIA; IncFIB; **IncFII**
RB12	RB12-74 T-KPC-IncN-IncX1-KPC-108 K-N	aph(3′)-IIa; blaKPC-2; rmtB	IncFII; IncN; IncX1

### Detection of plasmid incompatibility factors

WeFaceNano identified all of the expected incompatibility factors, including multiple (2–4) incompatibility factors per plasmid in 8/22; 36% of cases (Table 2). When multiple incompatibility factors were present, they were frequently found to be members of the IncF incompatibility group. Compared to the original publication, two additional incompatibility factors were identified of which one was present in plasmid RB04-234 K-Flye.

## Results summary page

**Fig. 4:**
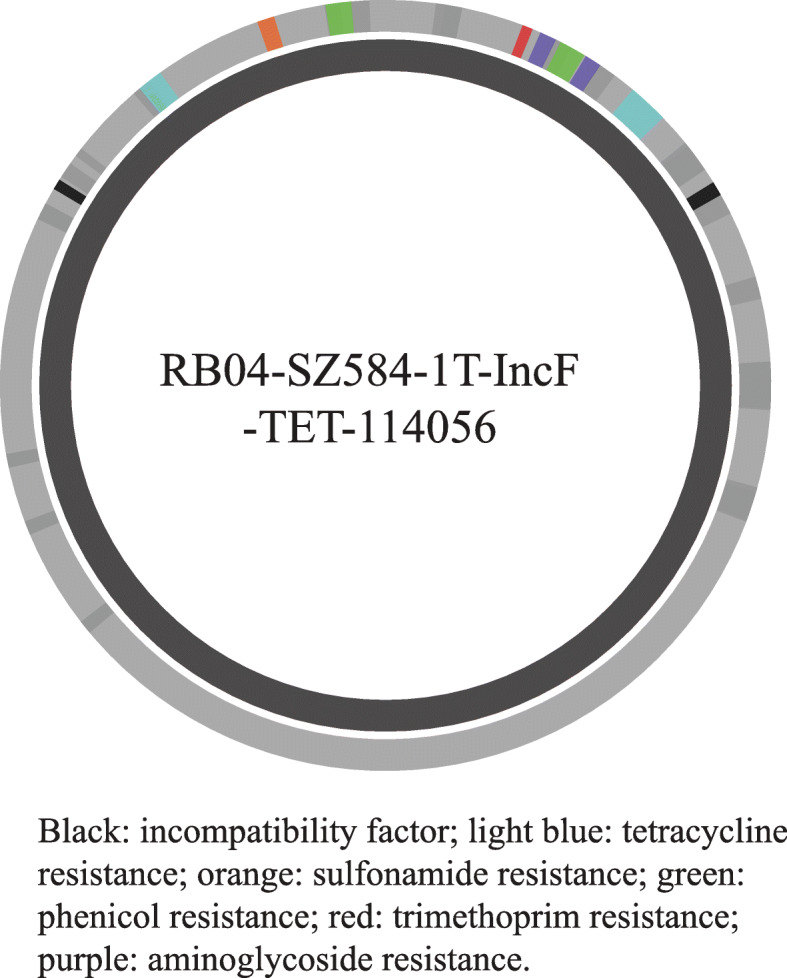
Visualization of a BLAST alignment. A ring image that represents the BLAST results of a metaFlye assembled plasmid from sample RB04. The inner ring indicates the top BLAST hit with the name of the identified plasmid in the middle, the outer ring indicates the assembled contig. Incompatibility factor are shown in black the other colors represent the antibiotic resistance genes

To show the output that is generated by the WeFaceNano application, a representative overview of the read statistics report and analysis results is given [16]. The read statistics report contains information about the uploaded raw reads and comprises several plots. The analysis results include an assembly table, an incompatibility factors table and an AMR gene table. The BLAST results are visualized in circular interactive web images with an inner circle that represents the top BLAST hit and an outer circle that represents the newly aligned contig. The locations of the antibiotic resistance genes and the incompatibility factors are indicated on the outer contig circle (Fig. 4, [16]). The colour of the incompatibility factors is black; all other colours represent the different antibiotic resistance genes detected.

## Discussion

We developed and validated WeFaceNano, an easy-to-use assembly and analysis pipeline for long plasmid sequence reads generated by the ONT platform. Two assemblers, Miniasm and Flye, were tested, and assembly and further analysis results were compared to previously published assembly and analysis results of 12 plasmid DNA extracts that contained multiple multidrug resistance-encoding plasmids [32]. Overall, similar results were obtained when the results of WeFaceNano was compared to previously published results, indicating that WeFaceNano performs comparably. Upon comparing different assemblers and assembly settings, we observed that the Flye assembler of WeFaceNano with the meta option (metaFlye) generated the most complete plasmids including a 234,535 kb plasmid that was only partly assembled when using other Flye settings or another assembler present within WeFaceNano, and in the original publication.

A major advantage of WeFaceNano is that it has an accessible, user-friendly interface and, after installation by a bioinformatician or system administrator, does not require knowledge of bioinformatics or programming. Additionally, the application is fast (average run time of ~ 10–15 min per sample), multiple relevant analyses are performed consecutively and informative reports, plots and additional visualizations are generated. The choice of tools within an application is critical for performance and accuracy. Among other tools, WeFaceNano contains MiniMap, Miniasm, and Racon, tools that have been demonstrated to be fast and accurate with relatively low memory usage compared to similar tools such as BWA-MEM, Canu and Nanopolish [7, 15]. The assembler Canu is more accurate than Miniasm by itself, but in combination with Racon polishing, the accuracy of Miniasm approaches the accuracy of Canu [7]. Initially, Canu was incorporated in WeFaceNano, but in our experience, in agreement with others [7], Canu was severely slower (assembly times of up to several days) and highly resource insensitive. After subsequent testing, we decided to exchange Canu for Flye, a long and error-prone read assembler software that was benchmarked against several other assemblers and generated better or comparable assemblies while being very fast [15, 26, 34].

It should be noted that the quality of the assembly relies on several factors. The quality and amount of the input DNA, the library kit used, the sequence coverage, and assembly parameters and options chosen may influence the final result and interpretation.

## Conclusions

To our knowledge, there currently is no application available for the assembly and analysis of long and error-prone plasmid reads that is comparable to WeFaceNano. New and promising algorithms and pipelines such as SLR, Nanopipe, doepipeline, CCBGpipe and NanoDJ [17, 21, 22, 24, 25] have been generated and made available but the use of these algorithms and pipelines almost always requires the support of bioinformatics and do not include features such as the identification, localization and visualization of AMR genes and plasmid incompatibility factors, that are relevant for a clinical microbiology setting.

## Availability and requirements

- Project name: WeFaceNano
- The Source code of WeFaceNano is available at: https://github.com/ErasmusMC-Bioinformatics/WeFaceNano
- Operating system(s): Unix (Platform independent with Docker)
- Programming language: Python
- License: GNU GPL v3.
- Any restrictions to use by non-academics: Not applicable.

## Data Availability

• The dataset analyzed during the current study is available in the GigaScience DataBase (GigaDB) data repository at: http://gigadb.org/dataset/100387 • The dataset generated during this study (assemblies) is available in the Zenodo data repository at https://zenodo.org/record/4719805#.YIbeMJAzaUk, doi:10.5281/zenodo.4719805 • A representative overview of a WeFaceNano read statistics and analysis report is available at: https://erasmusmc-bioinformatics.github.io/WeFaceNano/

## Abbreviations

AMR: anti-microbial resistance
BLAST: Basic Local Alignment Search Tool
DNA: deoxyribonucleic acid
.: incompatibility factor
(k)bp: (kilo)base pair
NCBI: National Center for Biotechnology Information; nr/nt database: protein/nucleotide database
ONT: Oxford Nanopore Technologies
SVG file: scalable vector graphics file
WGS: whole genome sequencing
